# Drought tolerance mechanisms across C3 and C3–C4 intermediate photosynthetic types revealed by physiological and gene expression profiling

**DOI:** 10.1038/s41598-025-33094-4

**Published:** 2026-01-11

**Authors:** Rabab H. M. Mohamed, Reem Badr, Amani Abdel-Latif, Ahmed Sorour

**Affiliations:** Department of Botany and Microbiology, Faculty of Science, Alexandria University, Moharam Bek, Alexandria, 21511 Egypt

**Keywords:** Drought stress, Oxidative stress, Antioxidant enzymes, Osmolyte accumulation, P5CS, sHSP26

## Abstract

Abiotic stress, particularly drought, significantly reduces crop yields and threatens global agricultural sustainability. This study investigated drought and recovery responses in four plant species with contrasting photosynthetic types: *Triticum aestivum* (C3), *Helianthus annuus* (C3), *Chenopodium album* (intermediate-C4), and *Alternanthera brasiliana* (C4-like). Drought markedly reduced plant fresh biomass (up to 80% in *H. annuus*) and relative water content, particularly in *C. album*. Oxidative damage intensified, with *H. annuus* showing the greatest increase in hydrogen peroxide (258%) and *C. album* exhibiting the highest malondialdehyde accumulation (284%). Antioxidant enzymes were strongly activated; catalase activity increased dramatically in *C. album* (837%) and *H. annuus* (630%). Proline levels increased sharply, particularly in *T. aestivum* and *C. album*, indicating enhanced osmotic adjustment. Carotenoid content also rose significantly in *H. annuus* (141%), suggesting photoprotective adaptation. Gene expression analysis revealed upregulation of *TaP5CS* in *T. aestivum*, correlating with proline accumulation, and *CaHSP26* in *C. album*, potentially stabilizing photosystem II. Principal component analysis identified catalase activity, root-to-shoot ratio, hydrogen peroxide, and proline as major contributors to drought response variance. These findings highlight species-specific strategies for drought tolerance and recovery, with C3 species showing strong enzymatic and osmotic adjustments, and intermediate-C4 and C4-like species exhibiting greater tissue integrity and ROS balance. This comparative framework provides valuable insights for developing drought-resilient crops.

**Supplementary Information:**

The online version contains supplementary material available at 10.1038/s41598-025-33094-4.

## Introduction

Abiotic stress remains the leading cause of crop failure, reducing average yields of major crops by more than 50% and threatening global agricultural sustainability. Among abiotic factors, drought is particularly detrimental, affecting approximately 33% of the world’s agricultural land and significantly limiting plant growth and productivity. Roughly one-third of the global land area is categorized as arid or semi-arid, and projections suggest that by 2050, over half of all agricultural land will be subject to drought stress^1^. The impact of drought on plant performance is multifactorial, depending on the developmental stage, genetic background, stress duration, and severity.

Drought stress impairs water uptake by decreasing water potential at the root-soil interface, triggering physiological and molecular responses. One of the primary responses is stomatal closure, which restricts transpiration and gas exchange, leading to reduced stomatal conductance and photosynthesis^2^. Concurrently, mesophyll tissue experiences turgor loss and pigment degradation, resulting in reduced relative water content (RWC), diminished chlorophyll levels, and overall biomass decline. Water deficit also promotes the accumulation of reactive oxygen species (ROS), such as hydrogen peroxide (H_2_O_2_), which compromise membrane integrity through lipid peroxidation, increasing malondialdehyde (MDA) levels^3^. To mitigate oxidative damage, plants activate a complex antioxidant defense system composed of enzymatic components such as superoxide dismutase (SOD), catalase (CAT), and peroxidases (POX), which detoxify ROS^4^. In addition, compatible osmolytes such as proline soluble sugars, and soluble proteins contribute to osmotic adjustment and maintenance of turgor^5^. These osmoprotectants also include amino acids sugars, and tertiary amines, with proline being the most commonly accumulated under stress^6^. However, its quantitative contribution to osmotic adjustment remains debated^7^. Phenolic compounds have also gained attention for their antioxidant roles, including metal ion chelation, ROS scavenging, and modulation of oxidative enzymes. These metabolites exhibit redox activity, acting as hydrogen donors, reducing agents, and singlet oxygen quenchers, thereby enhancing plant tolerance under stress conditions^8^.

Four plant species were selected for this study based on their contrasting photosynthetic features, growth forms, and ecological strategies: *Helianthus annuus* L. (sunflower), a C3 dicot of agricultural and bioenergy significance with moderate drought tolerance^9^; *Triticum aestivum* (wheat), a globally important C3 monocot staple crop^10^; *Chenopodium album*, a medicinally and nutritionally valuable species considered a C3–C4 intermediate^11^; and *Alternanthera brasiliana*, a stress-resilient herbaceous dicot, with C4-like traits reported within its genus^12,13^. Although a true C4 species was not included, this selection captures key aspects of physiological diversity relevant to carbon fixation efficiency, stomatal anatomy, and drought adaptation strategies.

To investigate molecular responses associated with drought and recovery in these species, we selected three stress-responsive genes as molecular markers, guided by the species’ functional classification and physiological plasticity. Two genes—*TaP5CS* (from wheat) and its putative homolog *HaP5CS* (from sunflower)—encode Δ^1^-pyrroline-5-carboxylate synthase, a bifunctional enzyme with glutamate kinase and γ-glutamyl phosphate reductase activity, which catalyzes a key step in the proline biosynthesis pathway^14^. Proline functions as an osmoprotectant under drought conditions, contributing to osmotic adjustment, membrane stabilization, and detoxification of reactive oxygen species (ROS)^15^. Upregulation of *P5CS* has been strongly associated with increased proline accumulation and enhanced drought tolerance in wheat^3,16^, while overexpression studies further confirm its functional relevance in stress adaptation^16^. Transcriptomic evidence suggests that *HaP5CS* may play similar roles in sunflower, particularly under water-deficit conditions^17^. In contrast, *CaHSP26* from *Chenopodium album* was selected to represent an alternative molecular defense mechanism, reflecting the species’ evolutionary classification as a C3–C4 intermediate and its physiological plasticity under abiotic stress. This gene encodes a small heat shock protein (sHSP) localized in the thylakoid lumen of chloroplasts, where it protects photosystem II (PSII) from stress-induced denaturation, facilitates protein folding, and cooperates with antioxidant systems to mitigate ROS damage. Its broad responsiveness to drought, heat, and oxidative stress underscores its utility as a molecular marker for stress resilience in *C. album*^18^. The choice to study *CaHSP26* instead of *P5CS* in this species reflects a deliberate focus on capturing alternative, species-specific molecular strategies underlying drought adaptation. Molecular analysis was not conducted on *Alternanthera brasiliana* due to the lack of annotated gene sequences and limited genomic data in public repositories such as GenBank, which posed significant constraints on candidate gene identification and primer design.

Understanding how plants regulate antioxidant defenses and osmotic balance, and how specific stress-responsive genes contribute to drought tolerance and recovery, is key to identifying genotypes with improved resilience. Therefore, this study aimed to: (i) assess the effects of drought and subsequent re-watering on physiological, biochemical, and anatomical traits in four species representing distinct stress strategies; (ii) evaluate oxidative stress and antioxidant enzyme responses during the vegetative stage using integrated physiological and biochemical measurements; and (iii) investigate the expression of *TaP5CS*, *HaP5CS*, and *CaHSP26* as representative markers of osmotic adjustment and protein stability mechanisms, thereby providing a comparative framework for understanding drought tolerance and recovery in plants with diverse photosynthetic and functional traits.

## Results

### Effects of drought and recovery on growth parameters

**Fig. 1:**
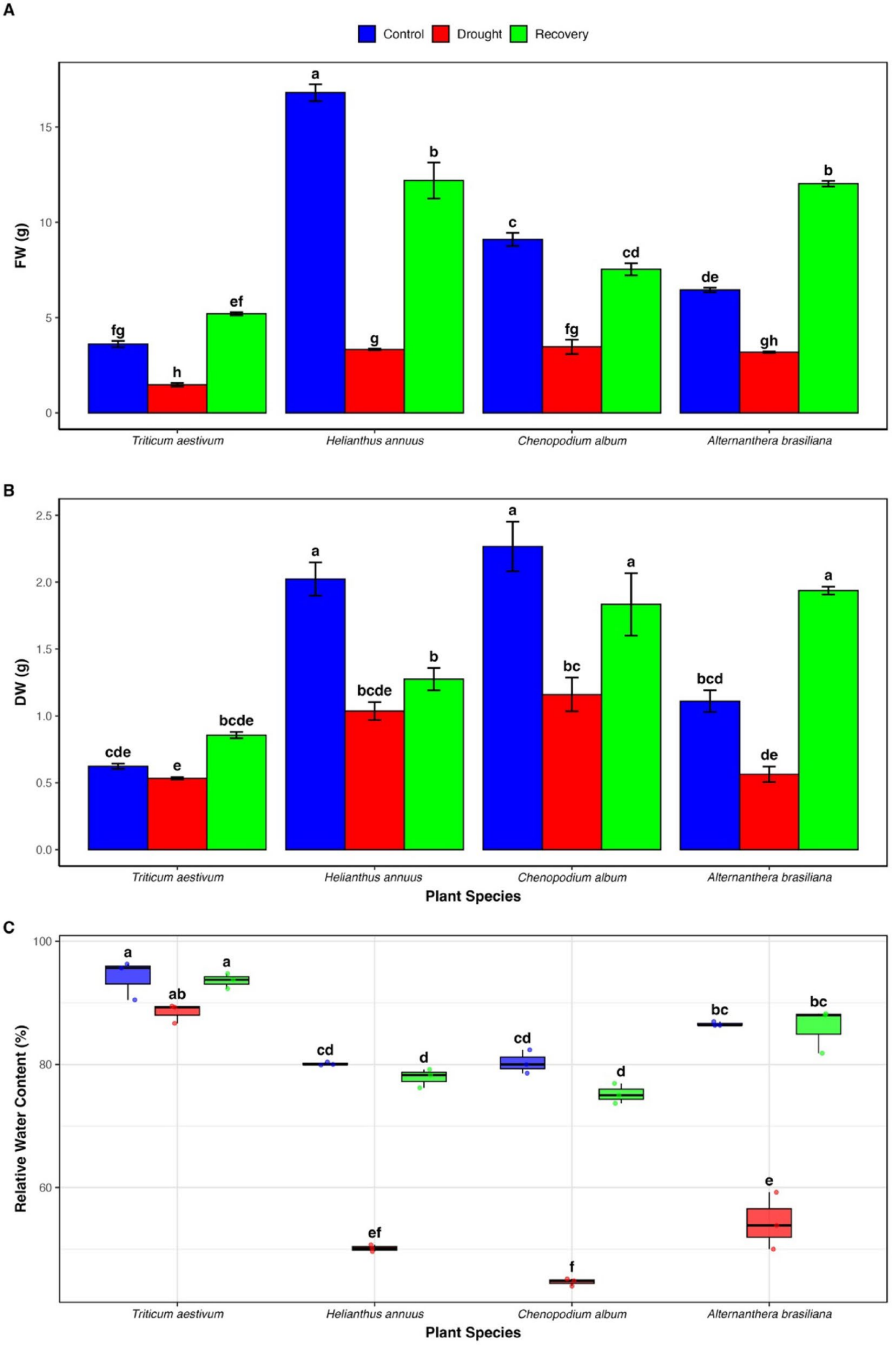
Effects of drought and recovery on biomass and water status in four plant species. Fresh weight (FW), dry weight (DW), and relative water content (RWC) were assessed in *Triticum aestivum, Helianthus annuus, Chenopodium album, and Alternanthera brasiliana* (n = 3) under control, drought, and recovery treatments. (**A**, **B**) FW and DW are shown as bar graphs representing mean ± SD. (C) RWC is shown as boxplots; boxes represent the interquartile range (25th–75th percentile), the line inside each box indicates the median, and whiskers represent the 5th and 95th percentiles. Statistical significance was determined by two-way ANOVA followed by Tukey’s post hoc test (*P* < 0.05). Different letters denote significant differences between treatments within each species.

The effects of drought on fresh and dry weights of *H. annuus*, *T. aestivum*, *C. album*, and *A. brasiliana* are shown in Fig. 1. All species experienced significant reductions in fresh weight under drought conditions. The most pronounced decrease was observed in *H. annuus* (80.2%), while *A. brasiliana* exhibited the least reduction (50.7%). The declines in *T. aestivum* (59.3%) and *C. album* (61.9%) were comparable. In terms of dry weight, drought caused significant reductions in all species except *T. aestivum*, where the decrease was not statistically significant.

Re-watering improved both fresh and dry weights in all species. Notably, *T. aestivum* and *A. brasiliana* exceeded their control fresh weight values during recovery, with *T. aestivum* reaching 5.20 g compared to 3.61 g under control conditions. Their dry weights also surpassed control levels post-recovery. Although the recovered fresh weights of *H. annuus* and *C. album* increased relative to drought-stressed plants, they did not reach control values.

Root-to-shoot ratio (R/S) increased significantly under drought in all species, with the greatest increase observed in *H. annuus* (109.1%). After re-watering, R/S declined but remained higher than control levels in all species. (Table S1) Relative water content (RWC) declined significantly in *A. brasiliana* (37.2%), *H. annuus* (37.4%), and *C. album* (44.4%) under drought, while the reduction in *T. aestivum* (6%) was not statistically significant. Re-watering restored RWC to near-control levels in all species (Fig. 1).

### Oxidative stress markers and antioxidant enzyme activities

**Fig. 2:**
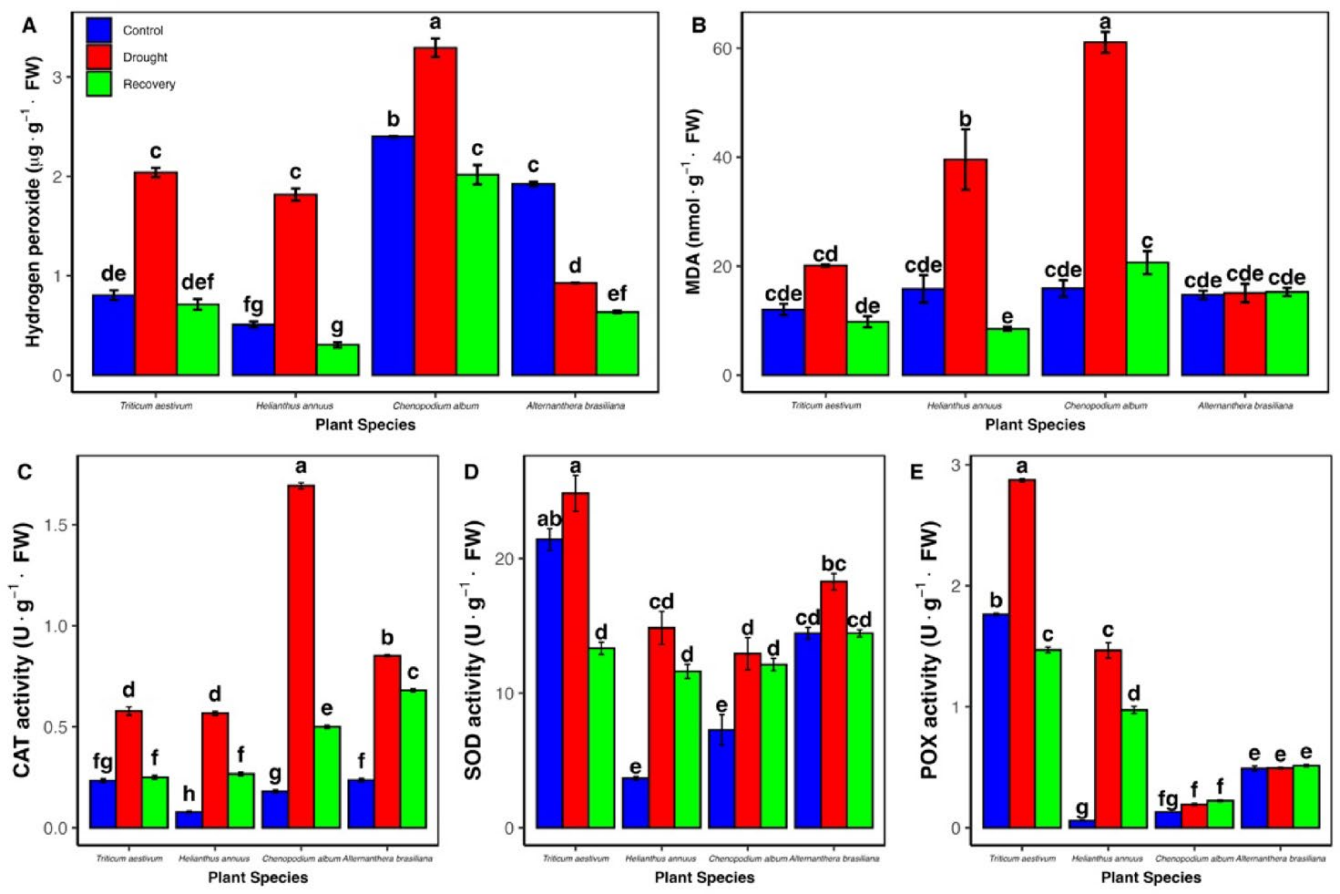
Oxidative stress markers and antioxidant enzyme activities in four plant species under drought and recovery conditions. (**A**) Hydrogen peroxide (H_2_O₂) content, (**B**) malondialdehyde (MDA) content, (**C**) catalase (CAT) activity, (**D**) superoxide dismutase (SOD) activity, and (**E**) peroxidase (POX) activity in leaves of *Triticum aestivum, Helianthus annuus, Chenopodium album, and Alternanthera brasiliana*. Bars represent means (± SD, n = 3). Different letters above bars indicate significant differences between means (*P* < 0.05) according to Tukey’s post hoc test following two-way ANOVA.

Drought stress induced oxidative damage, as evidenced by increased levels of hydrogen peroxide (H_2_O_2_) and malondialdehyde (MDA) in most species (Fig. 2). *H. annuus* showed the greatest increase in H_2_O_2_ (257.8%), followed by *T. aestivum* (153.7%) and *C. album* (37.2%). Interestingly, *A. brasiliana* exhibited a notable decline in H_2_O_2_ under drought (to 0.927 µg/g FW), with a further decrease upon recovery (0.635 µg/g FW), well below its control level (1.92 µg/g FW).

MDA levels increased in all species under drought, reflecting enhanced lipid peroxidation. The most substantial increase was seen in *C. album* (283.8%), followed by *H. annuus* (150.4%), *T. aestivum* (67%), and *A. brasiliana* (2.2%). It is important to note that all species established a low basal MDA level under control conditions, with *A. brasiliana* exhibiting the lowest change, demonstrating superior membrane protection under stress.

The activities of antioxidant enzymes catalase (CAT), superoxide dismutase (SOD), and peroxidase (POX) were evaluated under drought (Fig. 2). CAT activity increased in all species under drought, with *C. album* showing the highest increase (837.3%), followed by *H. annuus* (629.6%), *A. brasiliana* (260.9%), and *T. aestivum* (147.7%). SOD activity also rose, most notably in *H. annuus* (303.9%), followed by *C. album* (77.9%), *A. brasiliana* (26.5%), and *T. aestivum* (16.0%). POX activity exhibited the most pronounced increase in *H. annuus* (2371.9%), followed by *T. aestivum* (62.9%), while *C. album* (48.7%) and *A. brasiliana* (0.7%) showed minimal changes. Enzyme activities generally declined after re-watering, approaching or exceeding control levels in most species; however, in *T. aestivum*, activities returned to or dropped slightly below control levels.

### Osmolyte accumulation and total phenolics

**Fig. 3:**
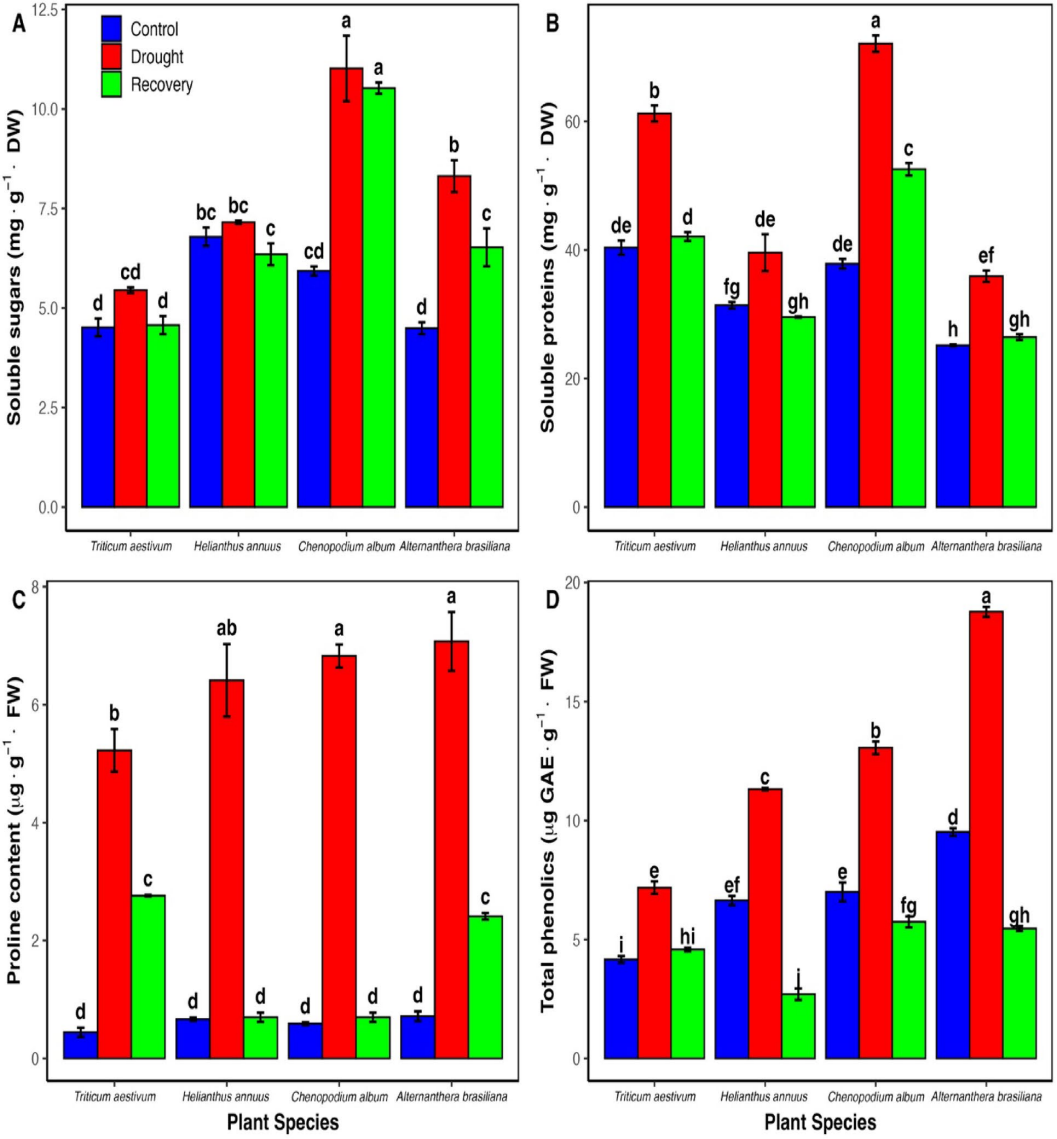
Osmolyte and total phenolic accumulation in four plant species under drought stress and recovery. (**A**) Soluble sugars content, (**B**) soluble proteins content, (**C**) proline content, and (**D**) total phenolics content in *Triticum aestivum, Helianthus annuus, Chenopodium album, and Alternanthera brasiliana*. Bars represent means (± SD, n = 3). Different letters above bars indicate significant differences between means (*P* < 0.05) according to Tukey’s post hoc test following two-way ANOVA.

Soluble protein levels increased significantly in all species under drought, followed by a decline upon recovery. *C. album* showed the highest increase (90.4%). Proline content increased markedly in response to drought, with *T. aestivum* showing the highest elevation (1078.6%), followed by *C. album* (1057.2%), *A. brasiliana* (889.4%), and *H. annuus* (863.9%). Although proline levels decreased after re-watering, they remained higher than control values. Soluble sugar content followed a similar trend to proteins and proline, increasing under drought and decreasing during recovery, but not fully returning to control levels (Fig. 3).

Drought treatment led to increased phenolic accumulation in all species (Fig. 3), with the highest rise observed in *A. brasiliana* (97.3%), followed by *C. album* (86.6%), *T. aestivum* (72.6%), and *H. annuus* (70.5%). Under control conditions, *A. brasiliana* exhibited the highest phenolic levels (9.52 µg/g FW), while *T. aestivum* had the lowest (4.16 µg/g FW). Following re-watering, phenolic levels declined in all species except *T. aestivum*, where values remained elevated compared to the control, suggesting a sustained secondary metabolite response.

### Photosynthetic pigments

Drought induced species-specific alterations in chlorophyll and carotenoid contents (Table S2). Total pigment levels increased significantly in *H. annuus* (59.3%), driven by marked rises in chlorophyll a (41.6%), chlorophyll b (70.7%), and carotenoids (141.1%), resulting in a reduced chlorophyll a/b ratio. In contrast, pigment levels declined by 21–31% in *T. aestivum*, *C. album*, and *A. brasiliana*. Although *T. aestivum* also exhibited a notable increase in carotenoids (41.5%) under drought, pigment levels in all species generally returned to control values upon recovery. An exception was *A. brasiliana*, which showed a post-recovery increase in total pigments to 9.17 mg/g FW—approximately 46.5% higher than its drought level (6.26 mg/g FW) and 12% above the control value (8.17 mg/g FW).

### Leaf anatomy: stomata, wax deposition, trichomes, and stomatal morphometric data

**Fig. 4:**
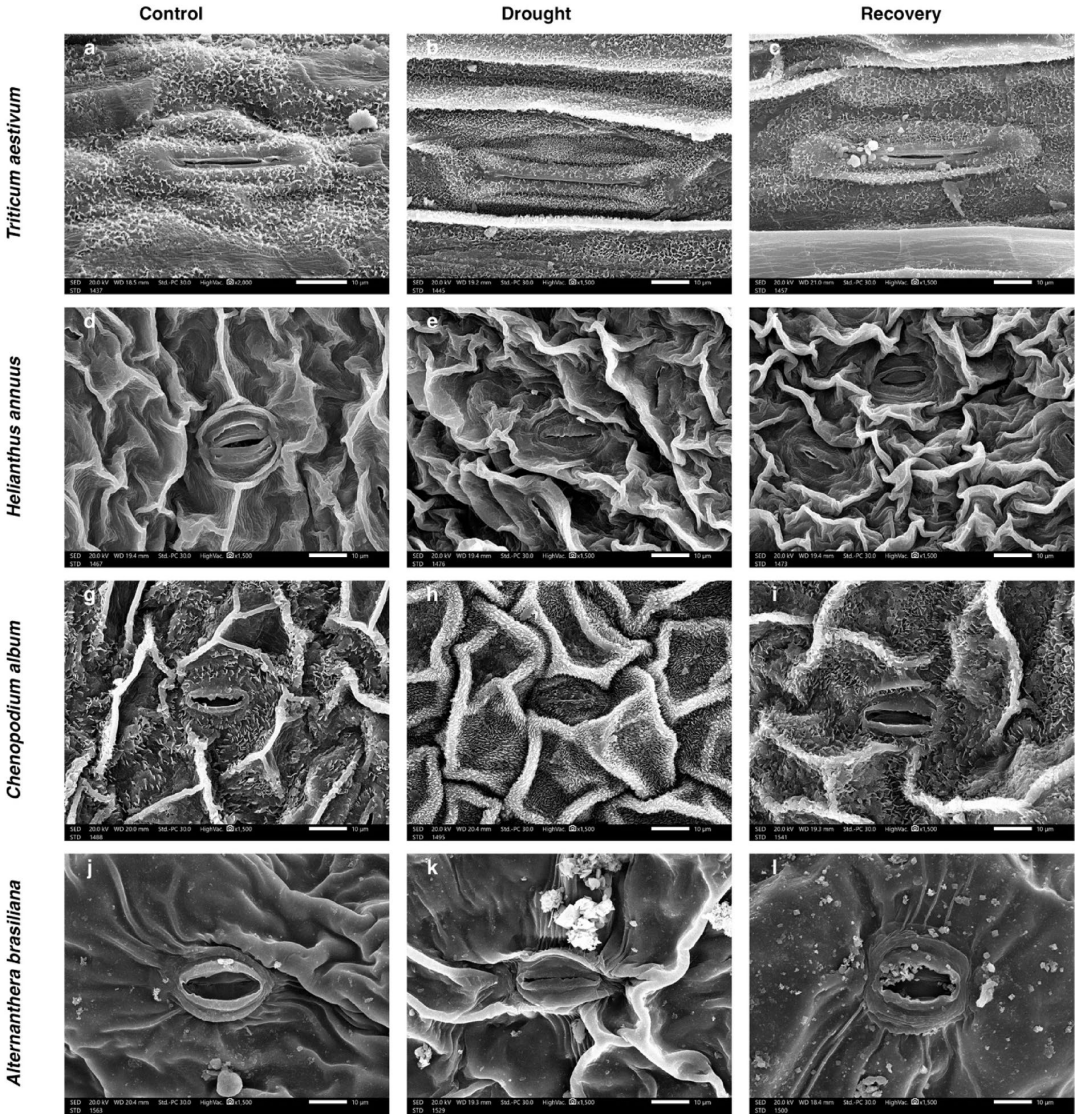
Scanning electron microscopy (SEM) analysis of adaxial leaf epidermis in four plant species under control, drought, and recovery conditions. Representative SEM micrographs show stomatal and epidermal surface features of *Triticum aestivum* (**a**–**c**), *Helianthus annuus* (**d**–**f**), *Chenopodium album* (**g**–**i**), and *Alternanthera brasiliana* (**j**–**l**) under control, drought, and recovery treatments, respectively. Drought stress visibly altered stomatal aperture, wax deposition, and epidermal integrity in all species. Magnifications and scale bars are indicated on each panel.

Under drought stress, notable changes were observed in leaf surface features and stomatal morphology across species. Scanning electron microscopy revealed increased epicuticular wax deposition on *C. album* and *T. aestivum* leaves, while *A. brasiliana* maintained relatively smooth surfaces (Fig. 4). Drought also induced the development of prominent trichomes and enhanced wax accumulation in *T. aestivum* (Figure S1).

Drought stress induced significant changes in stomatal pore dimensions across all four species, reflecting stomatal regulation under water deficit (Table S3). In *Triticum aestivum*, SPL increased markedly under drought (41.49 ± 2.29 µm) compared to control (21.83 ± 1.81 µm), while SPW decreased to zero, indicating complete stomatal closure. Partial reopening was observed during recovery, with SPL and SPW reaching 30.83 ± 2.99 µm and 1.22 ± 0.05 µm, respectively. *Helianthus annuus* showed no significant change in SPL between treatments, but SPW declined significantly under drought (0.41 ± 0.04 µm) relative to control (3.23 ± 0.73 µm), followed by partial reopening during recovery (1.22 ± 0.3 µm). In *Chenopodium album*, drought reduced both SPL (11.08 ± 0.05 µm) and SPW (0 ± 0 µm) compared to control, but both parameters increased significantly during recovery, with SPL reaching 18.22 ± 0.15 µm and SPW 3.4 ± 0.04 µm. *Alternanthera brasiliana* exhibited increased SPL under drought (18.66 ± 0.05 µm vs. 17.03 ± 0.06 µm in control) and the highest SPW during recovery (6.94 ± 1.13 µm), indicating a strong recovery in stomatal opening capacity. These patterns reveal species-specific stomatal adjustments to drought and rehydration.

### Gene expression patterns under drought

**Fig. 5:**
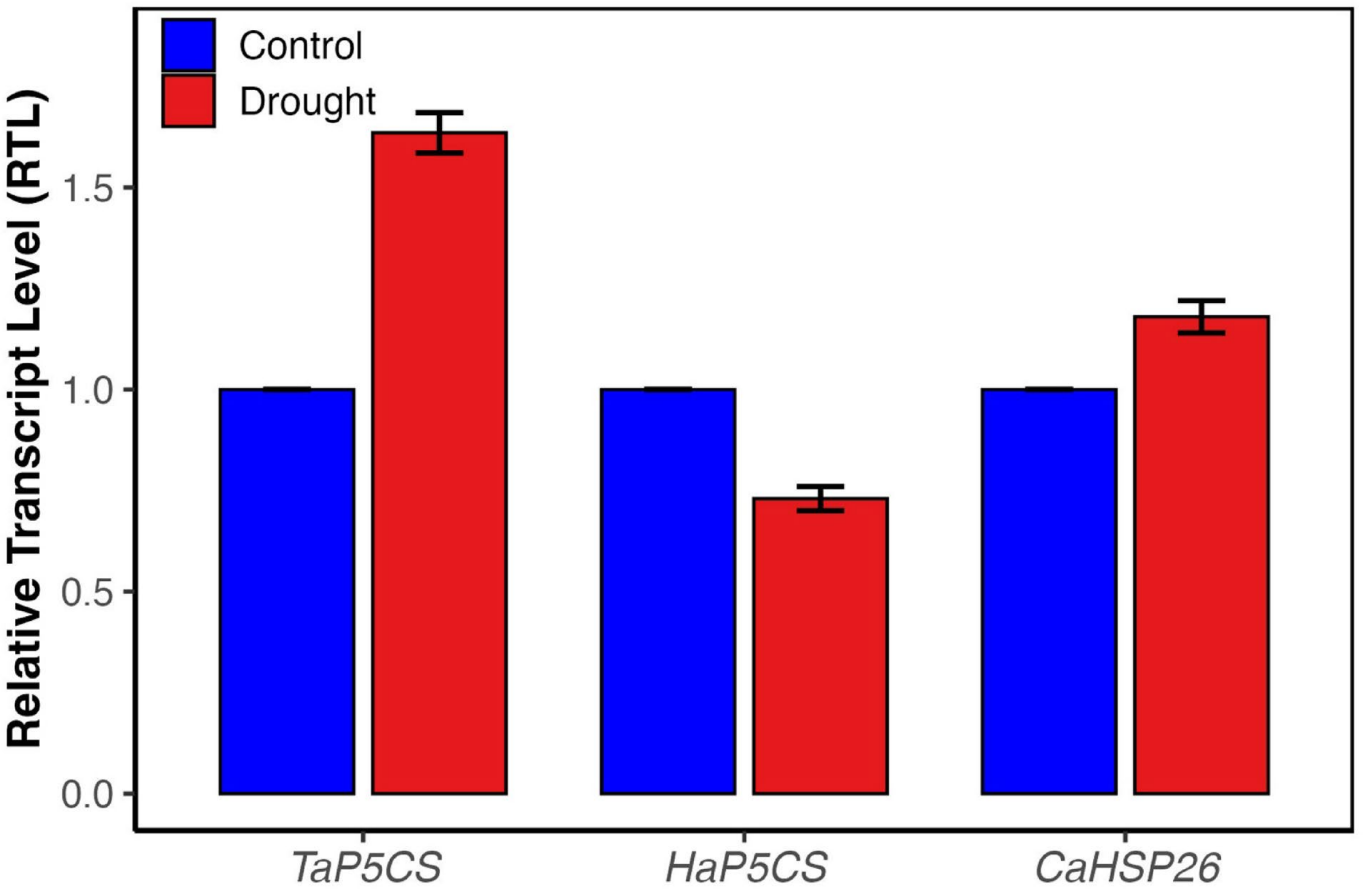
Relative transcript levels of stress-responsive genes in three plant species under control and drought treatments. Transcript levels of *TaP5CS, HaP5CS*, and *CaHSP26* were quantified by qRT-PCR in *Triticum aestivum, Helianthus annuus,* and *Chenopodium album,* respectively. Bars represent means ± SD (n = 2). Note: Molecular analysis was limited to the drought treatment stage due to technical constraints in primer stability across the recovery phase, focusing on genes activated during the immediate stress response."

Relative expression levels of selected drought-related genes showed species-specific responses (Fig. 5). In *T. aestivum* (C3), *TaP5CS* was upregulated (~ 1.6-fold) under drought, indicating enhanced proline biosynthesis for osmotic adjustment. In contrast, *H. annuus* (C3) showed a downregulation (~ 0.73-fold) of *HaP5CS*, suggesting a distinct regulatory response in proline metabolism. *C. album* (C3-C4 intermediate) exhibited a moderate induction of *CaHSP26* (~ 1.18-fold), implying a potential protective function of heat shock proteins in response to drought-induced stress. These expression profiles underscore divergent molecular mechanisms employed by the studied species in coping with water deficit.

### Principal component analysis of integrated drought and recovery responses

**Fig. 6:**
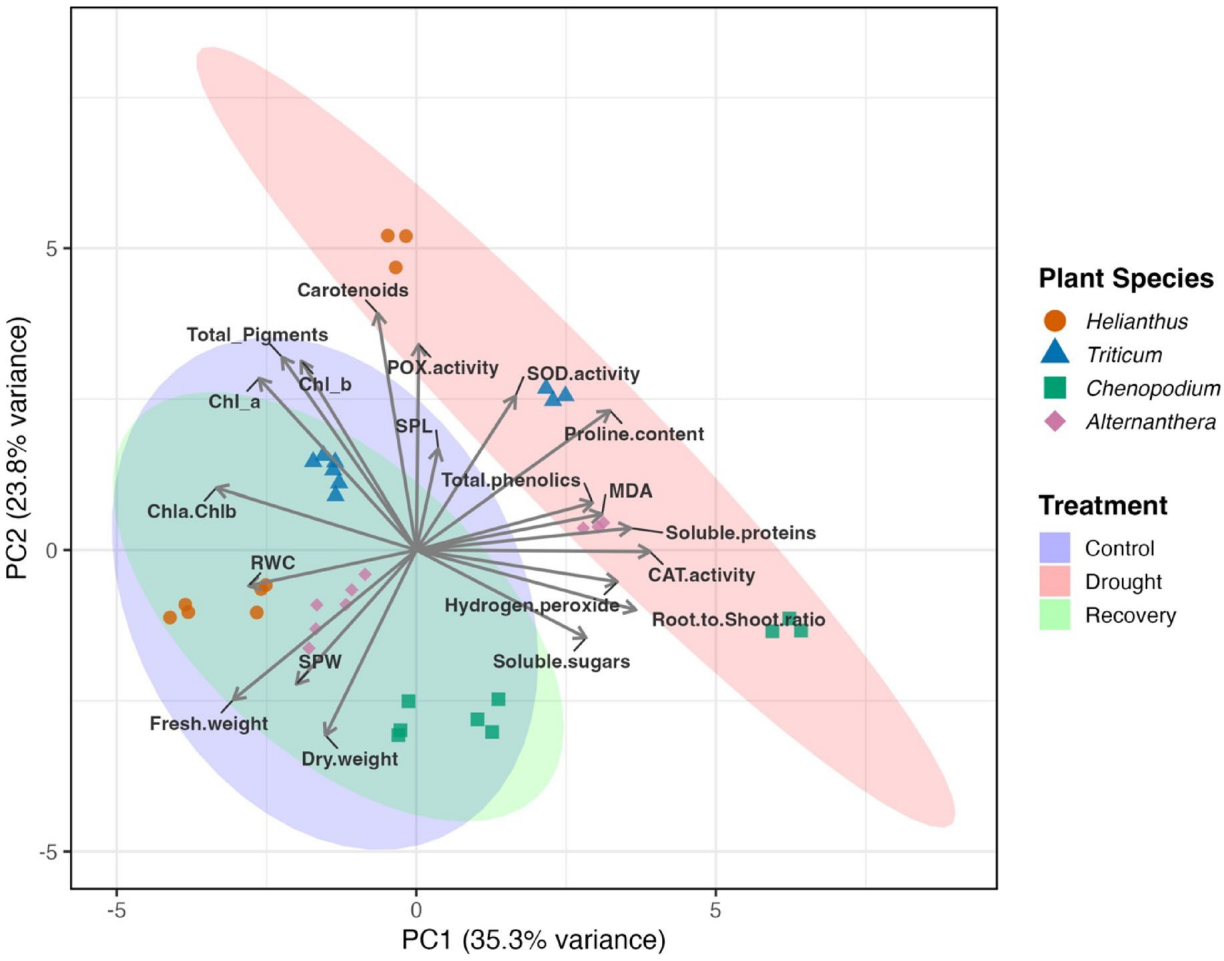
Principal Component Analysis (PCA) of physiological, biochemical, and anatomical traits in four plant species under drought and recovery. The biplot displays individual observations grouped by Plant Species (color and shape) and Treatment (ellipses), based on the first two principal components. These components explain 35.3% (PC1) and 23.8% (PC2) of the total variance, respectively. Vectors (arrows) representing the variable loadings are included, illustrating the contribution of each measured physiological, biochemical, and anatomical trait to the overall variance structure. The biplot visually links sample clusters to their underlying adaptive drivers, illustrating treatment effects and species-specific differences in multivariate trait patterns related to drought and recovery responses.

Principal Component Analysis (PCA) was conducted to explore multivariate patterns among physiological, biochemical, anatomical, and gene expression traits across species and treatments (Fig. 6). The first two principal components together explained 59.1% of the total variance**,** with PC1 accounting for 35.3% and PC2 for 23.8% (Figure S2).

PC1 primarily captured drought-induced variation, clearly separating drought-stressed samples from controls and recovery treatments (Table S5). Traits contributing most strongly to PC1 included catalase (CAT) activity (10.6%), root-to-shoot ratio (9.4%), soluble proteins (9.0%), hydrogen peroxide (7.9%), Chl a/b ratio (7.8%), proline (7.3%), and MDA (6.7%) (Table S4). These highlight the central role of oxidative stress markers, osmolyte accumulation, and biomass partitioning in shaping drought responses. Additional contributors included fresh weight (6.5%), total phenolics (6.1%), and soluble sugars (5.6%).

PC2 explained species-level variation and recovery dynamics. The traits with the highest contributions to PC2 were carotenoids (16.0%), peroxidase (POX) activity (12.1%), chlorophyll b (10.2%), and total pigments (10.6%) (Table S4), suggesting a strong role for photosynthetic pigment composition and antioxidant enzyme activity in differentiating species-specific responses. Other contributors included dry weight (9.8%), chlorophyll a (8.4%), fresh weight (6.4%), and superoxide dismutase (SOD) activity (6.8%), reflecting variation in pigment retention, stress recovery, and photoprotection.

Overall, the PCA identified oxidative stress mitigation, osmotic regulation, and biomass allocation as the dominant axes of drought-induced variance (PC1), while pigment dynamics and antioxidant enzyme activity contributed more to species-specific recovery responses and photosynthetic adjustments (PC2).

These multivariate patterns highlight the diverse physiological and biochemical mechanisms underlying drought response and recovery across species with distinct photosynthetic pathways.

## Discussion

Drought remains a major abiotic stress limiting plant productivity, contributing to up to 70% of global crop yield losses^19^. Its severity and frequency are expected to intensify under ongoing climate change, exacerbating soil moisture deficits and threatening global food security^20^. In the present study, all four species exhibited marked reductions in total plant fresh weight and relative water content (RWC) under drought stress (Fig. 1), indicative of impaired growth and disturbed plant–water relations. *Helianthus annuus* experienced the most substantial decline in fresh weight, while *Alternanthera brasiliana* showed the least reduction. Reductions in *Triticum aestivum* and *Chenopodium album* were comparable. Although dry weight decreased in most species, the reduction in *T. aestivum* was not statistically significant, suggesting a degree of structural resilience. Similarly, RWC decreased notably in *C. album*, *H. annuus*, and *A. brasiliana*, signaling considerable turgor loss and compromised water retention^21,22^. In contrast, *T. aestivum* exhibited only a slight and statistically non-significant decline in RWC, indicating better water conservation under stress. Upon re-watering, fresh weight improved in all species, with *T. aestivum* and *A. brasiliana* exceeding control levels, suggesting efficient recovery mechanisms and possible overcompensatory growth.

In response to water limitation, plants typically shift resource allocation toward root development to enhance water foraging, which is reflected in an increased root-to-shoot ratio (R/S)^23^. Drought elevated R/S in all species (Table S1), most prominently in *H. annuus*, indicating adaptive plasticity in biomass partitioning under stress. While R/S ratios declined post-rehydration, they remained elevated above control values, indicating partial retention of stress-induced architectural modifications, potentially due to physiological priming^24,25^.

A key manifestation of drought stress is oxidative damage, primarily driven by excess reactive oxygen species (ROS) such as hydrogen peroxide (H_2_O_2_). The resulting lipid peroxidation was evidenced by elevated malondialdehyde (MDA) levels across all species (Fig. 2). *C. album* showed the greatest MDA accumulation, while *A. brasiliana* exhibited minimal MDA elevation and a reduction in H_2_O_2_ levels, suggesting more effective ROS regulation and a stronger antioxidative defense. These interspecific differences in oxidative markers underscore the varying efficacy of ROS detoxification strategies^26,27^. Plants mitigate oxidative damage through a hierarchical antioxidant enzyme system in which superoxide dismutase (SOD) catalyzes the dismutation of superoxide radicals into H2O_2_, which is subsequently detoxified by catalase (CAT) and peroxidase (POX)^28^. Our results clearly point to distinct, species-specific antioxidant strategies for managing drought-induced oxidative stress across the C3-C4 intermediate types (Fig. 2). *Helianthus annuus* (C3) appears to rely heavily on a massive activation of POX (2371.9% increase) and SOD (303.9% increase) to rapidly detoxify ROS. In contrast, *Chenopodium album* (C3-C4 intermediate) utilized an exceptional increase in CAT activity (837.3% increase) as its primary line of defense against elevated H_2_O_2_. Meanwhile, *Triticum aestivum* (C3) relied on a moderate, coordinated activation across all enzymes. Most notably, the C4-like species, *Alternanthera brasiliana*, maintained relatively stable H_2_O_2_ and showed minimal increases in MDA and POX activity, suggesting a fundamental distinction in its ROS management, likely tied to its C4-like traits that enhance water-use efficiency and suppress photorespiratory ROS generation.

Following rehydration, enzyme activities generally declined to or below control levels, especially in *Triticum aestivum*, although some remained elevated in other species, possibly indicating stress memory or physiological priming.^29–31^. It is important to note that this decline is the expected physiological outcome. Since the substrate (H_2_O_2_) is rapidly depleted post-rehydration, the subsequent downregulation of CAT and POX activity signals a successful metabolic re-establishment and a return toward homeostasis, rather than a failure of the antioxidative system. The CAT response in *C. album* is consistent with its proposed role as a key H_2_O_2_ scavenger in stress-resilient species ^32^.

It is important to note that the observed general decline in CAT and POX activity during the recovery stage, despite reduced H2O_2_ and MDA levels, is the expected physiological outcome. Once the drought stress is alleviated and ROS levels fall, the intense enzymatic scavenging required during active stress is downregulated. This reduction in enzyme activity signals a successful metabolic re-establishment and a return toward homeostasis, rather than a failure of the antioxidative system.

Osmotic adjustment, via the accumulation of compatible solutes such as proline, soluble proteins, and sugars (Fig. 3), plays a pivotal role in drought tolerance by preserving cell turgor, stabilizing macromolecules, and scavenging ROS^15,33^. In our study, proline levels increased dramatically under drought, particularly in *T. aestivum* and *C. album*—consistent with its reported drought resilience^22^, followed by *A. brasilianan* and *H. annuus*. Soluble protein content also increased significantly under drought, most notably in *C. album*, reflecting enhanced metabolic activity and stress-related protein synthesis. Soluble sugar levels followed a similar trend, increasing in all species during drought and partially declining after rehydration. While post-recovery values for proline and sugars remained numerically above control in most cases, not all species showed statistically significant differences, reflecting differential recovery dynamics and possibly a short-term osmotic priming effect or delayed metabolic reversion^29^.

In parallel, total phenolic content—well-established ROS scavengers contributing to stress tolerance^34^—increased under drought in all species. The highest increase was observed in *A. brasiliana*, followed by *C. album*, *T. aestivum*, and *H. annuus*. Among controls, *A. brasiliana* exhibited the highest basal phenolic levels, while *T. aestivum* had the lowest, suggesting inherent differences in antioxidant capacity. After re-watering, phenolic content declined in all species. In *T. aestivum*, levels remained slightly higher than the control, though not statistically significant, suggesting a transient upregulation of phenolic metabolism during early recovery (Fig. 3). This pattern may reflect a short-lived stress memory or delayed downregulation of secondary metabolism as plants transition back to homeostasis, consistent with reports that phenolic responses can persist briefly during recovery as part of stress priming or residual defense activation^29^. These coordinated biochemical responses—including elevated osmolytes and phenolics—underscore species-specific drought tolerance mechanisms and recovery potential, with *T. aestivum* and *C. album* exhibiting particularly robust osmotic and antioxidative adjustments ^35,36^.

Photosynthetic pigment content, particularly chlorophyll and carotenoids, is sensitive to drought stress and plays a vital role in plant adaptation. In our study, chlorophyll levels decreased under drought in *T. aestivum,* C*. album*, and *A. brasiliana*, indicating impaired photosynthetic capacity. In contrast, *H. annuus* showed a substantial increase in chlorophyll a and chlorophyll b, leading to a net increase in total pigments and a reduced chlorophyll a/b ratio, suggesting a potential shift toward enhanced light-harvesting under stress. Carotenoids, which are known to contribute to photoprotection by scavenging ROS and protecting photosystems from oxidative damage^37^, increased significantly in *H. annuus* and *T. aestivum* during drought. These changes highlight the role of pigment modulation in maintaining photostability under oxidative stress conditions^38^. Following rehydration, pigment levels in most species returned to near-control values. However, A*. brasiliana* exhibited a notable increase in total pigments post-recovery, representing a significant rise compared to its drought value and above its control level. This enhanced pigment recovery may indicate a compensatory mechanism or a delayed activation of pigment biosynthesis during recovery.

Leaf anatomical changes also contributed to drought responses. Stomatal pore width (SPW) significantly declined in all species, with complete closure observed in *T. aestivum* and *C. album*, minimizing water loss^39,40^. Recovery of SPW occurred in C*. album* and A*. brasiliana*, while *H. annuus* showed minimal reopening. Meanwhile, stomatal pore length (SPL) increased under drought in *T. aestivum* and *A. brasiliana*, possibly indicating structural adaptation without aperture widening. These interspecific differences in stomatal behavior highlight distinct strategies of water-use efficiency and stress recovery^41–45^. Additional traits such as increased cuticular wax deposition in *T. aestivum* and *C. album*, and enhanced trichome density in *T. aestivum*, *H. annuus*, and *C. album*, may further reduce water loss through reflective and barrier functions^46,47^.

At the molecular level, drought tolerance involves the regulation of stress-responsive genes, including those encoding osmoprotectants and molecular chaperones. Our data demonstrate species-specific expression of small heat shock protein *sHSP26* and proline biosynthesis gene *P5CS*. In *T. aestivum*, the upregulation of *TaP5CS* coincided with elevated proline accumulation and enhanced antioxidant enzyme activity, indicating a coordinated response involving osmoprotection and reactive oxygen species (ROS) detoxification—hallmarks of drought responses in C₃ plants^48^. In contrast, *HaP5CS* was downregulated in *Helianthus annuus*, suggesting a divergent drought adaptation strategy that may rely on alternative osmolytes or signaling pathways. This is consistent with recent findings in sunflower, where drought stress induced the accumulation of soluble sugars and proteins, rather than proline, indicating a shift in osmoprotectant profiles^17,49^. Additionally, in *Chenopodium album*, the chloroplast-targeted *CaHSP26* appears to stabilize photosystem II components under drought-induced oxidative stress, as evidenced by increased SOD activity and reduced ROS accumulation. This supports its proposed role as a molecular chaperone that safeguards photosynthetic efficiency under abiotic stress^50,51^.

PCA provided a robust multivariate perspective on species-specific physiological, biochemical, and anatomical responses to drought and recovery. The first two components captured 59.1% of the total variance, with PC1 (35.3%) reflecting general treatment-induced responses (drought vs. control/recovery) and PC2 (23.8%) distinguishing species-level variation and recovery strategies. The high contribution of oxidative stress markers (CAT activity, H_2_O_2_, MDA) and osmotic regulators (proline, soluble proteins) to PC1 (Table S4) strongly supports and integrates the univariate findings, confirming the centrality of these classical mechanisms in drought defense. ^15,52^. Conversely, PC2 was driven by photoprotective traits such as carotenoids and POX activity (Table S4), validating the divergence of species-specific strategies during stress and recovery. These PC2-driving variables clearly separate the C3 species from the C3–C4 intermediate/C4-like species (Table S5), thus demonstrating that photosynthetic/functional type strongly dictates the recovery trajectory. This pattern is consistent with prior reports emphasizing the importance of pigment regulation and ROS detoxification in stress tolerance^37,38^.

In summary, the PCA revealed that drought tolerance and recovery are underpinned by coordinated shifts in oxidative stress defense, osmolyte accumulation, pigment metabolism, and anatomical plasticity. These multivariate insights not only confirm the univariate trends observed in physiological markers but also provide a systems-level perspective on how plants with distinct photosynthetic pathways diverge in their adaptive strategies.

Despite these robust, coordinated findings across the physiological, biochemical, and anatomical levels, there are inherent limitations to this comparative study. The molecular analysis was intentionally focused on three core pathway markers (*P5CS* and *HSP*) to capture key mechanisms (osmotic adjustment and protein stability) but does not delineate the full complexity of species-specific regulatory networks. Furthermore, due to technical constraints involving the stability of reference genes across diverse species and multiple treatments, gene expression analysis was limited to the drought-stressed stage, excluding the recovery phase. Future research endeavors should employ deep sequencing techniques, such as RNA-Seq, to capture the full transcriptome of drought-responsive genes, including *LEA* (Late Embryogenesis Abundant proteins) and other critical gene families. This will provide a comprehensive map of the diverse molecular strategies employed by these contrasting photosynthetic types and fully detail the mechanisms of recovery and stress memory.

Collectively, our results highlight that plant responses to drought and subsequent recovery involve species-specific coordination of physiological, biochemical, anatomical, and molecular traits. C3 species such as *T. aestivum* and *H. annuus* showed strong activation of enzymatic antioxidant systems and osmotic adjustment but differed in their recovery efficiency. In contrast, the intermediate-C4 *C. album* and C4-like *A. brasiliana* demonstrated greater maintenance of tissue integrity and ROS balance, supporting the idea that photosynthetic type and associated anatomical traits influence stress resilience. The persistence of specific physiological, biochemical, and photoprotective responses during recovery—including elevated proline, phenolics, antioxidant enzymes, and pigment content—particularly in *T. aestivum*, *C. album*, and *A. brasiliana*, may reflect a form of transient stress memory or priming that enhances resilience to subsequent drought episodes^29^. Understanding these multifaceted and dynamic responses deepens our knowledge of plant adaptation to water deficits and provides a valuable framework for selecting or engineering drought-resilient crops in the context of global climate change.

## Methods

### Experimental design and sampling

Seeds of *Triticum aestivum* (wheat), *Helianthus annuus* (sunflower), and *Chenopodium album* were obtained from the Agricultural Research Center, Ministry of Agriculture, Giza, Egypt. These seeds are commercially available and distributed for research and agricultural use. *Alternanthera brasiliana* was propagated via softwood cuttings from plants maintained in the greenhouse and botanical collection of the Department of Botany, Faculty of Science, Alexandria University. No wild specimens were collected, and no permits were required. All plant materials were used in accordance with institutional and national guidelines. Uniform seeds were selected based on size, shape, and viability, then surface-sterilized with 0.1% Hg Cl_2_ for 1 min, rinsed thoroughly with sterile distilled water, and germinated on moist filter paper in Petri dishes at 28 °C in the dark for 3 days. Seedlings exhibiting similar morphology and well-developed roots were transplanted into 2-L pots containing sandy loam soil. All plants were grown under natural sunlight with temperatures of 25–30 °C during the day and 15–20 °C at night. Irrigation was applied every two days.

Drought stress was induced in 30-day-old plants by withholding water for seven days, followed by a seven-day recovery period with regular watering. The successful induction and maintenance of severe drought stress was physiologically confirmed by measuring the Relative Water Content (RWC) at the end of the 7-day period. Pots (three biological replicates per treatment) were arranged in the botanical garden of the Faculty of Science, Alexandria University. Fully expanded upper mature leaves were sampled from three plants per species for analysis. Whole plants were washed with sterile distilled water and gently blotted dry. Fresh weight (FW) was measured immediately, and dry weight (DW) was determined after drying samples in a hot-air oven at 70 °C until a constant weight was achieved.

### Relative water content (RWC)

RWC was determined following the Clarke and Mccaig method ^53^. Fresh leaf samples were weighed (FW), soaked in deionized water for 24 h at 4 °C to obtain the turgid weight (TW), and subsequently dried at 80 °C to a constant weight (DW). RWC was calculated as:

### Oxidative stress markers and antioxidant enzyme activities

Malondialdehyde (MDA) content, a marker of lipid peroxidation, was determined by mixing 0.5 ml of plant extract with 1 ml of 20% trichloroacetic acid (TCA) containing 0.5% thiobarbituric acid (TBA)^54^. Samples were incubated at 100 °C for 30 min, cooled on ice, and centrifuged at 10,000 g for 10 min. Absorbance was measured at 532 and 600 nm, and MDA concentration was calculated using an extinction coefficient of 155 mM⁻^1^ cm⁻^1^. H₂O₂ content was measured according to the method of Velikova et al.^55^.

Fresh leaf tissues (0.5 g) were homogenized in ice-cold extraction buffer (50 mM phosphate buffer, pH 7.0, containing 1% polyvinylpyrrolidone), and the homogenate was centrifuged at 12,000 g for 15 min at 4 °C. The supernatant was used for enzyme assays as described by Esfandiari et al. ^56^. Catalase (CAT) activity was measured following Aebi method ^57^ by monitoring the decomposition of hydrogen peroxide (H₂O₂) at 240 nm. One unit of CAT activity was defined as the amount of enzyme that decomposes 1 µmol of H₂O₂ per minute. Peroxidase (POX) activity was determined according to the method of Shannon et al.^58^, based on the oxidation of guaiacol in the presence of H₂O₂. The increase in absorbance at 470 nm due to tetraguaiacol formation was recorded, and enzyme activity was expressed in units per gram fresh mass. Superoxide dismutase (SOD) activity was estimated following the method of Beauchamp and Fridovich^59^ which measures the enzyme’s ability to inhibit the photochemical reduction of nitro blue tetrazolium (NBT). One unit of SOD activity was defined as the amount of enzyme required to cause 50% inhibition of NBT reduction at 560 nm.

### Determination of soluble proteins, free proline, total soluble sugars, and total phenolics

Soluble protein content was estimated according to the method of Rausch^60^, which is based on the Coomassie Brilliant Blue G-250 dye-binding technique. Fresh leaf tissues (0.5 g) were homogenized in extraction buffer, and the protein concentration was determined by measuring the absorbance at 595 nm. Bovine serum albumin (BSA) was used as the standard for the calibration curve, and results were expressed as mg g⁻^1^ fresh mass.

Free proline content was determined using the acid ninhydrin-based colorimetric method of Bates et al.^61^. Briefly, 0.5 g of fresh tissue was homogenized in 3% (w/v) sulfosalicylic acid. After centrifugation, the supernatant was mixed with acid ninhydrin reagent and glacial acetic acid, then heated at 100 °C for 1 h. The reaction mixture was extracted with toluene, and the absorbance of the toluene phase was measured at 520 nm. The concentration of proline was calculated from a standard curve and expressed as µmol g⁻^1^ fresh mass.

Total soluble sugars were quantified using the phenol–sulfuric acid method described by Dubois et al.^62^. A known volume of the ethanol extract from homogenized plant tissues was reacted with 5% phenol and concentrated sulfuric acid. The reaction mixture was incubated at room temperature, and absorbance was recorded at 490 nm. Glucose was used to generate a standard curve, and results were expressed as mg g⁻^1^ fresh mass.

Total phenolic content was estimated using a modified Folin–Ciocalteu method of Singleton and Rossi ^63^. Twenty microliters of plant extract were mixed with 1.58 ml distilled water and 100 µl Folin–Ciocalteu reagent. After 30 s to 8 min, 300 µl of 20% Na_2_CO_3_ was added. Samples were incubated at 40 °C for 20 min, and absorbance was measured at 750 nm. Results were expressed as µg gallic acid equivalents (GAE) per g fresh weight.

### Photosynthetic pigments

Leaf tissues were ground in a chilled mortar with 80% acetone. The homogenate was centrifuged, and the supernatant was stored at − 20 °C until spectrophotometric measurements. Chlorophyll a, chlorophyll b, and total carotenoids were quantified at wavelengths of 662, 645, and 470 nm, respectively, using the equations of Lichtenthaler and Wellburn^64^.

### Scanning electron microscopy (SEM)

Leaf samples from control, drought-stressed, and recovered plants were fixed in 4% formaldehyde and 1% glutaraldehyde (4F1G) in phosphate buffer (pH 7.2) at 4 °C for 3 h. Post-fixation was carried out in 2% osmium tetroxide (OsO₄) at 4 °C for 2 h. Samples were washed, dehydrated in a graded ethanol series, dried using the critical point method, mounted on aluminum stubs with carbon paste, and gold-coated (400 Å thickness). Observations were made using a JEOL JSM-5300 SEM operated at 15–20 kV^65^.

### RNA extraction, cDNA synthesis, and quantitative real-time PCR (qRT-PCR)

Total RNA was extracted from plant tissues using Biozol reagent (Bioer Technology, Japan) following the manufacturer’s protocol. RNA concentration and purity were determined by measuring absorbance at 260 nm and 280 nm with a NanoDrop 2000 spectrophotometer (Thermo Fisher Scientific, USA). Samples with an A260/A280 ratio between 1.8 and 2.0 were used for further analysis. One microgram of RNA was reverse transcribed into cDNA using the Viva cDNA synthesis kit (Vivantis, Singapore) according to the manufacturer’s instructions. Quantitative real-time PCR (qRT-PCR) reactions were performed using RealMOD™ Green W 2× qPCR mix (Catalog #25350, Intron Biotechnology, USA) in a final volume of 20 µl, which included 1 µl of cDNA template and 2 µl of primer mix (forward and reverse primers). Thermal cycling was conducted on a StepOnePlus™ Real-Time PCR System (Thermo Fisher Scientific, USA) with the following program: initial denaturation at 95 °C for 10 min; 40 cycles of denaturation at 95 °C for 20 s, annealing at 60 °C for 15 s, elongation at 72 °C for 60 s; followed by a final extension at 72 °C for 5 min^66^.

Relative gene expression levels were calculated using the 2^−ΔΔCt method by normalizing the Ct values of target genes to the geometric mean of the reference genes.

Target gene expression was normalized against two internal reference genes: elongation factor 1-alpha (*EF1α*) and 18S ribosomal RNA.

For *Triticum aestivum*, *EF1α* primers were: forward 5′-GGTTAAGATGATTCCCACCAAGCC-3′ and reverse 5′-GACAACACCAACAGCAACAGTCTG-3′. For *Helianthus annuus*, *EF1α* primers were: forward 5′-TGCCCAAGAAGTTGCTGGTG-3′ and reverse 5′-ACGTGCCCAGGTGAGTCGAT-3′. The *EF1α* primers for *Chenopodium album* were: forward 5′-CCGAGCGTGAACGTGGTAT-3′ and reverse 5′-TAGTACTTGGTGGTTTCGAATTTCC-3′. 18S rRNA primers were: forward 5′-TCCTGAGTAACGACGAGACC-3′ and reverse 5′-CACGATGAAATTTCCCCAAGAT-3′. The 18S rRNA primer set was applied universally due to the high conservation of the ribosomal gene across monocots, dicots, and C3-C4 intermediates, serving as a stable, fixed baseline for the geometric mean calculation across all comparative species.

Specific primers for the target genes were: *CaHSP26* (*Chenopodium album*): forward 5′-ATGGCAAGCAAGGGTATTACATGCAG-3′, reverse 5′-GGTGACAATGAGTCGATCAATCCAA-3′. *TaP5CS* (*Triticum aestivum*): forward 5′-ACAGAGATAAAGTAGCAGAGAC-3′, reverse 5′-AGACCTTCAACACCCACAG-3′. *HaP5CS* (*Helianthus annuus*): forward 5′-TGGTGGAGGACTTATCGGACT-3′, reverse 5′-TCCGAGGTAGCCAGTGTAGT-3′. The primers for *HaP5CS* were adopted from a previously published study of Chen et al.^67^, as no annotated *H. annuus* P5CS nucleotide sequence. Relative gene expression levels were calculated using the 2^ − ΔΔCt method by normalizing the Ct values of target genes to the geometric mean of the reference genes^68^. This robust normalization strategy, utilizing the geometric mean of the *EF1α* and 18S rRNA Ct values, serves as the necessary internal validation practice for comparative gene expression across treatments and diverse species.

### Statistical analysis

All experiments were performed using a completely randomized design with three biological replicates per treatment, except for gene expression analysis where two biological replicates were used. Data are presented as mean values ± standard deviation (SD). Statistical analyses were conducted using R software (version 4.3.1) within RStudio (version 2025.05.0 Build 496).

Two-way analysis of variance (ANOVA) was conducted to evaluate the effects of drought, species, and their interaction. When significant differences were detected (*P* < 0.05), multiple comparisons of means were performed using Tukey’s Honest Significant Difference (HSD) post hoc test.

Principal Component Analysis (PCA) was conducted to explore multivariate patterns across physiological, biochemical, and anatomical traits. Gene expression data were excluded from the PCA to prevent bias due to limited replication. Only numeric variables with complete data were included. Biplots were used to visualize the clustering of treatments and species, and trait loadings were examined to interpret the contributions of variables to each principal component.

## Supplementary Information

Below is the link to the electronic supplementary material.

Supplementary Material 1

## Data Availability

All data generated or analyzed during this study are included in this published article and its Supplementary Information files. Gene sequences used for qRT-PCR analysis were sourced as follows: Triticum aestivum P5CS (TaP5CS): GenBank accession KM523670.1 Chenopodium album HSP26 (CaHSP26): GenBank accession HQ012628.1 Helianthus annuus P5CS (HaP5CS): Primers adopted from Chen et al. (2024, Agronomy, 14, 22995; 10.3390/agronomy14122995). No annotated H. annuus P5CS nucleotide sequence was available in GenBank at the time of the study. No novel nucleotide sequences or transcriptomic datasets were generated in this study.

## References

[1] Ashraf, F. & Siddiqi, E. H. Mitigation of drought-induced stress in sunflower (*Helianthus annuus* L.) via foliar application of Jasmonic acid through the augmentation of growth, physiological, and biochemical attributes. *BMC Plant Biol.* **24**, 592 (2024). 38907232 10.1186/s12870-024-05273-4 PMC11193306

[2] Buckley, T. N. How do stomata respond to water status?. *New Phytol.* **224**, 21–36. 10.1111/nph.15899 (2019). 31069803 10.1111/nph.15899

[3] Xue, X. et al. Exogenous SNP alleviates drought stress in wheat during the grain-filling stage by modulating TaP5CS gene transcription. *Int. J. Mol. Sci.* **26**, 618 (2025). 39859332 10.3390/ijms26020618 PMC11765586

[4] Guo, M. et al. The plant heat stress transcription factors (HSFS): Structure, regulation, and function in response to abiotic stresses. *Front. Plant Sci.* **7**, 114 (2016). 26904076 10.3389/fpls.2016.00114 PMC4746267

[5] Verslues, P. E. & Sharma, S. Proline metabolism and its implications for plant–environment interaction. *Arabidopsis Book* **8**, e0140 (2010). 22303265 10.1199/tab.0140 PMC3244962

[6] Annunziata, M. G., Ciarmiello, L. F., Woodrow, P., Dell’aversana, E. & Carillo, P. Spatial and temporal profile of glycine betaine accumulation in plants under abiotic stresses. *Front. Plant Sci.* **10**, 230. 10.3389/fpls.2019.00230 (2019). 30899269 10.3389/fpls.2019.00230 PMC6416205

[7] Bohnert, H. J. & Sheveleva, E. Plant stress adaptations-making metabolism move. *Curr. Opin. Plant Biol.* **1**, 267–274 (1998). 10066591 10.1016/s1369-5266(98)80115-5

[8] Cheynier, V., Comte, G., Davies, K. M., Lattanzio, V. & Martens, S. Plant phenolics: Recent advances on their biosynthesis, genetics, andecophysiology. *Plant Physiol. Biochem.* **72**, 1–20 (2013). 23774057 10.1016/j.plaphy.2013.05.009

[9] Earley, A. M., Nolting, K. M., Donovan, L. A. & Burke, J. M. Trait variation and performance across varying levels of drought stress in cultivated sunflower (*Helianthus annuus* L.). *AoB Plants* **16**, plae031 (2024). 39011498 10.1093/aobpla/plae031 PMC11247526

[10] Zhang, J. et al. Effect of drought on agronomic traits of rice and wheat: A meta-analysis. *Int. J. Environ. Res. Public Health* **15**, 839 (2018). 29695095 10.3390/ijerph15050839 PMC5981878

[11] Yorimitsu, Y., Kadosono, A., Hatakeyama, Y., Yabiku, T. & Ueno, O. Transition from C3 to proto-Kranz to C3–C4 intermediate type in the genus Chenopodium (Chenopodiaceae). *J. Plant Res.* **132**, 839–855 (2019). 31473860 10.1007/s10265-019-01135-5 PMC7205854

[12] Gowik, U., Engelmann, S., Bläsing, O. E., Raghavendra, A. S. & Westhoff, P. Evolution of C4 phosphoenolpyruvate carboxylase in the genus Alternanthera: Gene families and the enzymatic characteristics of the C4 isozyme and its orthologues in C3 and C 3/C4 Alternantheras. *Planta* **223**, 359–368 (2006). 16136331 10.1007/s00425-005-0085-z

[13] da Silva, L. C. et al. Antimicrobial activity of *Alternanthera brasiliana* Kuntze (Amaranthaceae): A biomonitored study. *Lat. Am. J. Pharm.* **30**, 147–153 (2011).

[14] Furlan, A. L., Bianucci, E., Giordano, W., Castro, S. & Becker, D. F. Proline metabolic dynamics and implications in drought tolerance of peanut plants. *Plant Physiol. Biochem.* **151**, 566–578 (2020). 32320942 10.1016/j.plaphy.2020.04.010

[15] Szabados, L. & Savouré, A. Proline: A multifunctional amino acid. *Trends Plant Sci.* **15**, 89–97. 10.1016/j.tplants.2009.11.009 (2010). 20036181 10.1016/j.tplants.2009.11.009

[16] Du, L. et al. TaERF87 and TaAKS1 synergistically regulate TaP5CS1/TaP5CR1-mediated proline biosynthesis to enhance drought tolerance in wheat. *New Phytol.* **237**, 232–250 (2023). 36264565 10.1111/nph.18549

[17] Shen, J. et al. Physiology and transcriptomics highlight the underlying mechanism of sunflower responses to drought stress and rehydration. *iScience* **26**, 108112 (2023). 37860690 10.1016/j.isci.2023.108112 PMC10583116

[18] Haq, N. U. et al. Molecular characterization of *Chenopodium album* chloroplast small heat shock protein and its expression in response to different abiotic stresses. *Plant Mol. Biol. Rep.* **31**, 1230–1241 (2013).

[19] Yadav, S. et al. Effect of abiotic stress on crops, in *Sustainable Crop Production* (IntechOpen, 2020). 10.5772/intechopen.88434.

[20] Li, N. et al. Impacts of future climate change on rice yield based on crop model simulation—A meta-analysis. *Sci. Total Environ.* **949**, 175038 (2024). 39059663 10.1016/j.scitotenv.2024.175038

[21] Zhu, Y., Luo, X., Nawaz, G., Yin, J. & Yang, J. Physiological and Biochemical Responses of four cassava cultivars to drought stress. *Sci. Rep.* **10**, 6968 (2020). 32332812 10.1038/s41598-020-63809-8 PMC7181862

[22] Dahiya, B. & Chaudhry, S. Response of *Chenopodium album* L. to varying levels of water stress: Effects on physiological and biochemical parameters. *Plant Arch.* **20**, 2081–2086 (2020).

[23] Mahajan, S. & Tuteja, N. Cold, salinity and drought stresses: An overview. *Arch. Biochem. Biophys.* **444**, 139–158 (2005). 16309626 10.1016/j.abb.2005.10.018

[24] Seidel, S. J. et al. The overlooked effects of environmental impacts on root:shoot ratio in experiments and soil-crop models. *Sci. Total Environ.* **955**, 176738 (2024). 39389147 10.1016/j.scitotenv.2024.176738

[25] Zhou, G. et al. Drought-induced changes in root biomass largely result from altered root morphological traits: Evidence from a synthesis of global field trials. *Plant Cell Environ.* **41**, 2589–2599 (2018). 29879755 10.1111/pce.13356

[26] Zhang, J. & Kirkham, M. B. Antioxidant responses to drought in sunflower and sorghum seedlings. *New Phytol.* **132**, 361–373 (1996). 26763632 10.1111/j.1469-8137.1996.tb01856.x

[27] Cruz de Carvalho, M. H. Drought stress and reactive oxygen species. *Plant Signal. Behav.* **3**(3), 156–165 (2008). 19513210 10.4161/psb.3.3.5536 PMC2634109

[28] Sarker, U. & Oba, S. Catalase, superoxide dismutase and ascorbate-glutathione cycle enzymes confer drought tolerance of *Amaranthus tricolor*. *Sci. Rep.* **8**, 16496 (2018). 30405159 10.1038/s41598-018-34944-0 PMC6220278

[29] Lukić, N., Kukavica, B., Davidović-Plavšić, B., Hasanagić, D. & Walter, J. Plant stress memory is linked to high levels of anti-oxidative enzymes over several weeks. *Environ. Exp. Bot.* **178**, 104166 (2020).

[30] Ru, C., Hu, X., Chen, D. & Wang, W. Drought stimulus enhanced stress tolerance in winter wheat (*Triticum aestivum* L.) by improving physiological characteristics, growth, and water productivity. *Plant Physiol. Biochem.* **214**, 108906 (2024). 38986237 10.1016/j.plaphy.2024.108906

[31] Jacques, C., Salon, C., Barnard, R. L., Vernoud, V. & Prudent, M. Drought stress memory at the plant cycle level: A review. *Plants* **10**, 1873 (2021). 34579406 10.3390/plants10091873 PMC8466371

[32] Semwal, V. K. & Khanna-Chopra, R. Enhanced oxidative stress, damage and inadequate antioxidant defense contributes towards insufficient recovery in water deficit stress and heat stress combination compared to either stresses alone in *Chenopodium album* (Bathua). *Physiol. Mol. Biol. Plants* **26**, 1331–1339 (2020). 32647451 10.1007/s12298-020-00821-2 PMC7326836

[33] Jogawat, A. Osmolytes and their role in abiotic stress tolerance in plants, in *Molecular Plant Abiotic Stress: Biology and Biotechnology* 91–104 (Wiley, 2019). 10.1002/9781119463665.ch5.

[34] Sharma, A. et al. Response of phenylpropanoid pathway and the role of polyphenols in plants under abiotic stress. *Molecules* **24**, 2452. 10.3390/molecules24132452 (2019). 31277395 10.3390/molecules24132452 PMC6651195

[35] Forlani, G., Trovato, M., Funck, D., & Signorelli, S. Regulation of proline accumulation and its molecular and physiological functions in stress defence, in Hossain, M., Kumar, V., Burritt, D., Fujita, M., Mäkelä, P. (eds) *Osmoprotectant-Mediated Abiotic Stress Tolerance in Plants* (Springer, Cham, 2019). 10.1007/978-3-030-27423-8_3.

[36] Sa Ânchez, F. J., Manzanares, Â., De Andres, E. F., Tenorio, J. Â. L. & Ayerbe, L. Turgor maintenance, osmotic adjustment and soluble sugar and proline accumulation in 49 pea cultivars in response to water stress. *Field Crops Res.* **59**, 225–235 (1998).

[37] Farooq, M., Wahid, A., Kobayashi, N., Fujita, D. & Basra, S. M. A. Plant drought stress: Effects, mechanisms and management. *Agronomy for Sustainable Development* vol. 29 185–212 Preprint at 10.1051/agro:2008021 (2009).

[38] Ru, C., Hu, X., Chen, D., Wang, W. & Zhen, J. Photosynthetic, antioxidant activities, and osmoregulatory responses in winter wheat differ during the stress and recovery periods under heat, drought, and combined stress. *Plant Sci.* **327**, 111557 (2023). 36481364 10.1016/j.plantsci.2022.111557

[39] Lawson, T. & Vialet-Chabrand, S. Speedy stomata, photosynthesis and plant water use efficiency. *New Phytol.* **221**, 93–98. 10.1111/nph.15330 (2019). 29987878 10.1111/nph.15330

[40] Yavas, I. et al. Drought-induced changes in leaf morphology and anatomy: Overview, implications and perspectives. *Pol. J. Environ. Stud.* **33**, 1517–1530. 10.15244/pjoes/174476 (2024).

[41] Faralli, M., Matthews, J. & Lawson, T. Exploiting natural variation and genetic manipulation of stomatal conductance for crop improvement. *Curr. Opin. Plant Biol.* **49**, 1–7. 10.1016/j.pbi.2019.01.003 (2019). 30851622 10.1016/j.pbi.2019.01.003 PMC6692497

[42] Galmés, J., Medrano, H. & Flexas, J. Photosynthetic limitations in response to water stress and recovery in Mediterranean plants with different growth forms. *New Phytol.* **175**, 81–93 (2007). 17547669 10.1111/j.1469-8137.2007.02087.x

[43] Shahinnia, F. et al. Genetic association of stomatal traits and yield in wheat grown in low rainfall environments. *BMC Plant Biol.* **16**, 150 (2016). 27378125 10.1186/s12870-016-0838-9 PMC4932692

[44] Xu, Z., Jiang, Y., Jia, B. & Zhou, G. Elevated-CO_2_ response of stomata and its dependence on environmental factors. *Front. Plant Sci.* **7**, 657. 10.3389/fpls.2016.00657 (2016). 27242858 10.3389/fpls.2016.00657 PMC4865672

[45] Hetherington, A. M. & Woodward, F. I. The role of stomata in sensing and driving environmental change. *Nature* **424**, 901–908 (2003). 12931178 10.1038/nature01843

[46] Benz, B. W. & Martin, C. E. Foliar trichomes, boundary layers, and gas exchange in 12 species of epiphytic Tillandsia (Bromeliaceae). *J. Plant Physiol.* **163**, 648–656 (2006). 16545998 10.1016/j.jplph.2005.05.008

[47] Seufert, P. et al. Building a barrier: The influence of different wax fractions on the water transpiration barrier of leaf cuticles. *Front. Plant Sci.* **12**, 7660602 (2022). 10.3389/fpls.2021.766602 PMC8766326 35069622

[48] Dudziak, K. et al. Analysis of wheat gene expression related to the oxidative stress response and signal transduction under short-term osmotic stress. *Sci. Rep.* **9**, 2743 (2019). 30808876 10.1038/s41598-019-39154-w PMC6391441

[49] Sevgi, B. & Leblebici, S. Exogenous sucrose alleviates salt stress in sunflower (*Helianthus annuus* L.) and canola (*Brassica napus* L.) by modulating osmotic adjustment and antioxidant defense system. *Physiol. Mol. Biol. Plants*10.1007/s12298-025-01571-9 (2025). 40256277 10.1007/s12298-025-01571-9 PMC12006602

[50] Waters, E. R. & Vierling, E. Plant small heat shock proteins: Evolutionary and functional diversity. *New Phytol.* **227**, 24–37. 10.1111/nph.16536 (2020). 32297991 10.1111/nph.16536

[51] Benešová, M. et al. The physiology and proteomics of drought tolerance in Maize: Early stomatal closure as a cause of lower tolerance to short-term dehydration?. *PLoS ONE* **7**, e38017 (2012). 22719860 10.1371/journal.pone.0038017 PMC3374823

[52] Mittler, R. Oxidative stress, antioxidants and stress tolerance. *TRENDSin Plant Sci.* **7**, 405 (2002). 10.1016/s1360-1385(02)02312-9 12234732

[53] Clarke, J. M. & Mccaig, T. N. Evaluation of techniques for screening for drought resistance in wheat. *Crop Sci.* **22**, 503–506 (1982).

[54] Wang, C. et al. The effect of excess Zn on mineral nutrition and antioxidative response in rapeseed seedlings. *Chemosphere* **75**, 1468–1476 (2009). 19328518 10.1016/j.chemosphere.2009.02.033

[55] Velikova, V., Yordanov, I. & Edreva, A. Oxidative stress and some antioxidant systems in acid rain-treated bean plants protective role of exogenous polyamines. *Plant Sci*. **151** www.elsevier.com/locate/plantsci (2000).

[56] Esfandiari, E., Shekari, F., Shekari, F. & Manouchehr, E. The effect of salt stress on antioxidant enzymes’ activity and lipid peroxidation on the wheat seedling. *Not. Bot. Hort. Agrobot. Cluj* **35**, 48 (2007).

[57] Aebi, H. Catalase in vitro. *Methods Enzymol.* **105**, 121–126 (1984). 6727660 10.1016/s0076-6879(84)05016-3

[58] Shannon, L. M., Kay, E. & Lew, J. Y. Peroxidase Isozymes from Horseradish Roots. *J. Biol. Chem.* **241**, 2166–2172 (1966). 5946638

[59] Beauchamp, C. & Fridovich, I. Superoxide dismutase: Improved assays and an assay applicable to acrylamide Gels1. *Analyt. Biochem.* **44**, 276 (1971). 4943714 10.1016/0003-2697(71)90370-8

[60] Rausch, T. The estimation of micro-algal protein content and its meaning to the evaluation of algal biomass I. Comparison of methods for extracting protein. *Hydrobiologia* **78**, 237–251 (1981).

[61] Bates, L. S., Waldren, R. P. & Teare, I. D. Rapid determination of free proline for water-stress studies. *Plant Soil* **39**, 205–207 (1973).

[62] Dubois, M., Gilles, K. A., Hamilton, J. K., Rebers, P. A. & Smith, F. Colorimetric method for determination of sugars and related substances. *Anal. Chem.* **28**, 350–356 (1956).

[63] Singleton, V. L. & Rossi, J. A. Colorimetry of total phenolics with phosphomolybdic-phosphotungstic acid reagents. *RossiAm. J. Enol. Vitic.* **16**, 144–158 (1965).

[64] Lichtenthaler, H. K. & Wellburn, A. R. Determinations of total carotenoids and chlorophylls a and b of leaf extracts in different solvents. *Biochem. Soc. Trans.*10.1042/bst0110591 (1983).

[65] Bozzola, J. J., & Russell, L. D. *Electron Microscopy: Principles and Techniques for Biologists*. (Jones and Bartlett c1999, 1999).

[66] Sorour, A. A., Badr, R., Mahmoud, N. & Abdel-Latif, A. Cadmium and zinc accumulation and tolerance in two Egyptian cultivars (S53 and V120) of *Helianthus annuus* L. as potential phytoremediator. *Int. J. Phytoremediat.*10.1080/15226514.2024.2343842 (2024). 10.1080/15226514.2024.2343842 38644603

[67] Chen, F. et al. Physiological evaluation of salt tolerance in sunflower seedlings across different genotypes. *Agronomy* **14**, 2995 (2024).

[68] Livak, K. J. & Schmittgen, T. D. Analysis of relative gene expression data using real-time quantitative PCR and the 2-ΔΔCT method. *Methods* **25**, 402–408 (2001). 11846609 10.1006/meth.2001.1262

