# Supplementary material for: Drought tolerance mechanisms across C3 and C3–C4 intermediate photosynthetic types revealed by physiological and gene expression profiling
Source: Sci Rep. 2026 Jan 11;16:1329. doi: 10.1038/s41598-025-33094-4 (PMC12796193; doi:10.1038/s41598-025-33094-4)
Supplement: Supplementary file 1 — Supplementary Material 1 [file 41598_2025_33094_MOESM1_ESM.docx]

**Drought Tolerance Mechanisms across C3 and C3-C4 Intermediate Photosynthetic Types Revealed by Physiological and Gene Expression Profiling**

Rabab H. M. Mohamed^1^, Reem Badr^1^, Amani Abdel-Latif^1^, and Ahmed A. Sorour^1*^

^1^ Department of Botany and Microbiology, Faculty of Science, Alexandria University, Alexandria, Egypt.

*****To whom correspondence should be addressed**:** Ahmed A. Sorour, email: ahmed.sorour@alexu.edu.eg

Ahmed A. Sorour: ORCID: <https://orcid.org/0000-0001-7515-7350>

Reem Badr: ORCID: <https://orcid.org/0000-0002-6146-7080>

Rabab Hassan: ORCID: <https://orcid.org/0009-0004-2041-6406>

Amani Abdel-Latif: ORCID: <https://orcid.org/0000-0002-5821-4752>

**Address:** Department of Botany and Microbiology, Faculty of Science, Alexandria University, Moharam Bek 21511, Alexandria, Egypt

**Postal code:** 21511

**Table S1. Growth parameters of four plant species under control, drought, and recovery conditions**. Values represent means ± standard error (n = 3) for root length, shoot length, and root-to-shoot ratio in *Triticum aestivum*, *Helianthus annuus*, *Chenopodium album*, and *Alternanthera brasiliana* under control, drought, and recovery treatments.

| **Plant** | **Treatment** | **Root length (cm)** | **Shoot length (cm)** | **Root-to-Shoot ratio** |
| --- | --- | --- | --- | --- |
| *Triticum aestivum* | Control | 12.67 ± 0.29^ab^ | 33.13 ± 2.35^ab^ | 0.38 ± 0.03^cd^ |
|  | Drought | 14.90 ± 1.93^a^ | 31.40 ± 1.42^abc^ | 0.47 ± 0.05^bc^ |
|  | Recovery | 15.33 ± 0.91^a^ | 38.10 ± 1.59^a^ | 0.40 ± 0.04^cd^ |
| *Helianthus annuus* | Control | 2.30 ± 0.26^c^ | 22.40 ± 6.80^c^ | 0.11 ± 0.03^f^ |
|  | Drought | 5.10 ± 0.20^c^ | 22.53 ± 2.55^c^ | 0.23 ± 0.02^e^ |
|  | Recovery | 5.00 ± 0.61^c^ | 33.07 ± 5.08^ab^ | 0.15 ± 0.02^ef^ |
| *Chenopodium album* | Control | 3.17 ± 0.31^c^ | 6.90 ± 0.95^d^ | 0.46 ± 0.02^bcd^ |
|  | Drought | 4.13 ± 0.32^c^ | 7.03 ± 0.51^d^ | 0.59 ± 0.02^a^ |
|  | Recovery | 5.23 ± 1.70^c^ | 10.00 ± 1.90^d^ | 0.51 ± 0.08^ab^ |
| *Alternanthera brasiliana* | Control | 11.00 ± 1.10^b^ | 30.10 ± 1.06^abc^ | 0.36 ± 0.03^d^ |
|  | Drought | 13.37 ± 1.55^ab^ | 24.37 ± 4.00^bc^ | 0.55 ± 0.03^ab^ |
|  | Recovery | 13.43 ± 2.20^ab^ | 34.27 ± 2.65^a^ | 0.39 ± 0.05^cd^ |

Different letters within each species indicate statistically significant differences between treatments according to one-way ANOVA followed by Tukey’s HSD post hoc test (*P* < 0.05).

|  | **Pigment content (mg g⁻¹ FW)** | | | | | |
| --- | --- | --- | --- | --- | --- | --- |
| **Plant** | **Treatment** | **Chl a** | **Chl b** | **Total Pigments** | **Carotenoids** | **Chl a/b** |
| *Triticum aestivum* | Control | 6.37 ± 0.07^c^ | 1.96 ± 0.05e^f^ | 9.73 ± 0.12^c^ | 1.39 ± 0.05^cd^ | 3.25 ± 0.06^a^ |
|  | Drought | 3.09 ± 0.04^g^ | 1.63 ± 0.03^g^ | 6.68 ± 0.16^f^ | 1.97 ± 0.09^b^ | 1.9 ± 0.02f^g^ |
|  | Recovery | 6.09 ± 0.07^d^ | 2.1 ± 0.07^de^ | 9.53 ± 0.18^cd^ | 1.34 ± 0.04^cde^ | 2.9 ± 0.06^bc^ |
| *Helianthus annuus* | Control | 8.33 ± 0.03^b^ | 2.49 ± 0.1^b^ | 12.27 ± 0.13^b^ | 1.45 ± 0.03^c^ | 3.34 ± 0.13^a^ |
|  | Drought | 11.79 ± 0.14^a^ | 4.26 ± 0.17^a^ | 19.54 ± 0.42^a^ | 3.5 ± 0.11^a^ | 2.77 ± 0.08^c^ |
|  | Recovery | 5.3 ± 0.07^e^ | 1.76 ± 0.04^fg^ | 8.11 ± 0.12^e^ | 1.06 ± 0.02^f^ | 3.02 ± 0.04^b^ |
| *Chenopodium album* | Control | 2.81 ± 0.03^h^ | 1.35 ± 0.06^h^ | 5.2 ± 0.12^g^ | 1.04 ± 0.04^f^ | 2.08 ± 0.07^def^ |
|  | Drought | 2.17 ± 0.02^i^ | 1.09 ± 0.06^i^ | 4.08 ± 0.1^h^ | 0.81 ± 0.04^g^ | 2 ± 0.1^ef^ |
|  | Recovery | 2.73 ± 0.02^h^ | 1.28 ± 0.03^hi^ | 4.75 ± 0.08^g^ | 0.74 ± 0.03^g^ | 2.13 ± 0.05^de^ |
| *Alternanthera brasiliana* | Control | 4.69 ± 0.05^f^ | 2.22 ± 0.03^cd^ | 8.19 ± 0.12^e^ | 1.28 ± 0.04^de^ | 2.12 ± 0.01^de^ |
|  | Drought | 3.23 ± 0.04^g^ | 1.81 ± 0.07^fg^ | 6.26 ± 0.15^f^ | 1.23 ± 0.04^e^ | 1.78 ± 0.05^g^ |
|  | Recovery | 5.46 ± 0.07^e^ | 2.4 ± 0.06^bc^ | 9.17 ± 0.14^d^ | 1.31 ± 0.02^cde^ | 2.27 ± 0.04^d^ |

**Table S2. Photosynthetic pigments content in the leaves of four plant species under control, drought, and recovery conditions.** Values represent means ± standard error (n = 3). Chlorophyll *a* (Chl a), chlorophyll *b* (Chl b), carotenoids, and total pigments are expressed in mg g⁻¹ fresh weight (FW).

Different letters within each species indicate statistically significant differences between treatments according to one-way ANOVA followed by Tukey’s HSD post hoc test (*P* < 0.05).

**Table S3. Stomatal pore length (SPL) and Stomatal pore width (SPW) of four plant species under Control, Drought, and Recovery treatments.**
Mean values ± standard error (SE) of SPL (µm) and SPW (µm) are presented for *Triticum aestivum*, *Helianthus annuus*, *Chenopodium album*, and *Alternanthera brasiliana* across treatments.

| **Plant** | **Treatment** | **SPL (µm)** | **SPW (µm)** |
| --- | --- | --- | --- |
| *Triticum aestivum* | Control | 21.83 ± 1.81b | 1.6 ± 0.25a |
|  | Drought | 41.49 ± 2.29a | 0 ± 0b |
|  | Recovery | 30.83 ± 2.99b | 1.22 ± 0.05a |
| *Helianthus annuus* | Control | 13.98 ± 0.61a | 3.23 ± 0.73a |
|  | Drought | 12.65 ± 0.61a | 0.41 ± 0.04b |
|  | Recovery | 12.79 ± 0.29a | 1.22 ± 0.3b |
| *Chenopodium album* | Control | 13.17 ± 0.1b | 2.56 ± 0.1b |
|  | Drought | 11.08 ± 0.05c | 0 ± 0c |
|  | Recovery | 18.22 ± 0.15a | 3.4 ± 0.04a |
| *Alternanthera brasiliana* | Control | 17.03 ± 0.06b | 4.93 ± 0.98ab |
|  | Drought | 18.66 ± 0.05a | 1.17 ± 0.2b |
|  | Recovery | 18.79 ± 0.23a | 6.94 ± 1.13a |

Different letters within each species indicate statistically significant differences between treatments according to one-way ANOVA followed by Tukey’s HSD post hoc test (*P* < 0.05).

**Table S4. Contributions of Physiological, Biochemical, and Anatomical Variables to Principal Components 1 and 2.**

|  | Dim.1 | Dim.2 |
| --- | --- | --- |
| Fresh.weight | 6.5325939378206 | 6.40344936794811 |
| Dry.weight | 1.60817944389417 | 9.81427172942125 |
| RWC | 5.48112417825589 | 0.365961047178896 |
| ROOT.TO.SHOOT. RATIO | 9.39327149857928 | 1.02474646239357 |
| SPL | 0.0926959968395356 | 2.91650630620691 |
| SPW | 2.81200933926866 | 5.15393391576808 |
| Soluble.proteins | 8.95348537442105 | 0.13740615337454 |
| Soluble.sugars | 5.60793949024299 | 2.20389875008079 |
| Proline.content | 7.29955407028991 | 5.54335951509255 |
| MDA | 6.72460925785994 | 0.368370070302344 |
| Hydrogen.peroxide | 7.87863183650468 | 0.28970075934346 |
| Total.phenolics | 6.05021409247324 | 0.640572209962319 |
| POX.activity | 0.00115041557735723 | 12.0681538790097 |
| CAT.activity | 10.6336700492725 | 0.000662296399036833 |
| SOD.activity | 1.91436264555638 | 6.75415800600343 |
| Chl_a | 4.83114776357408 | 8.39758239044031 |
| Chl_b | 2.57886726845922 | 10.1823091182294 |
| Carotenoids | 0.293555281627716 | 16.026012351175 |
| Chla.Chlb | 7.79725238623223 | 1.09881545446909 |
| Total_Pigments | 3.51568567325058 | 10.6101302172011 |

**Table S5. Principal Component Scores of Individual Samples Based on PC1 and PC2.**

| Sample | PC1_Score | PC2_Score |
| --- | --- | --- |
| 1 | -1.72224913282724 | 1.46161620296907 |
| 2 | -1.54888336119289 | 1.56454482519398 |
| 3 | -1.3534266262697 | 1.45493785628859 |
| 4 | 2.48868785065001 | 2.55228077474389 |
| 5 | 2.28180383541428 | 2.47358243947874 |
| 6 | 2.16500946431377 | 2.67713308500737 |
| 7 | -1.34797912600182 | 0.900548972806411 |
| 8 | -1.38714553988985 | 1.32154146010159 |
| 9 | -1.2879258545622 | 1.10658017018143 |
| 10 | -3.80682599354482 | -1.03028507833 |
| 11 | -3.85694897292956 | -0.905084021941842 |
| 12 | -4.11140859267389 | -1.1184704544347 |
| 13 | -0.343886641263554 | 4.68204166430605 |
| 14 | -0.173933237897591 | 5.20215752065083 |
| 15 | -0.476100052106946 | 5.21060123423399 |
| 16 | -2.66322199632904 | -1.03559592884458 |
| 17 | -2.51265341731037 | -0.581668155732867 |
| 18 | -2.59136197820003 | -0.647426489619585 |
| 19 | -0.299489814385608 | -3.06836791433304 |
| 20 | -0.266156883501124 | -2.98952418243226 |
| 21 | -0.130455580897699 | -2.5058148209746 |
| 22 | 5.94178898559543 | -1.34467449747059 |
| 23 | 6.22431256115576 | -1.13715957596438 |
| 24 | 6.42167938782756 | -1.33487698065243 |
| 25 | 1.26537857441764 | -3.01727713471855 |
| 26 | 1.01776580141795 | -2.80850256740763 |
| 27 | 1.3714785536799 | -2.47468880821524 |
| 28 | -0.856610279087729 | -0.402772829679523 |
| 29 | -1.17449082486693 | -0.902191631883218 |
| 30 | -1.07830650388045 | -0.660263481035282 |
| 31 | 2.78541856339386 | 0.365139943786463 |
| 32 | 3.12304481413145 | 0.453883820371945 |
| 33 | 3.03513131144532 | 0.38472279888255 |
| 34 | -1.79342914561787 | -1.62897014029524 |
| 35 | -1.6608402200136 | -0.909362248534743 |
| 36 | -1.67776992819241 | -1.30833582650262 |


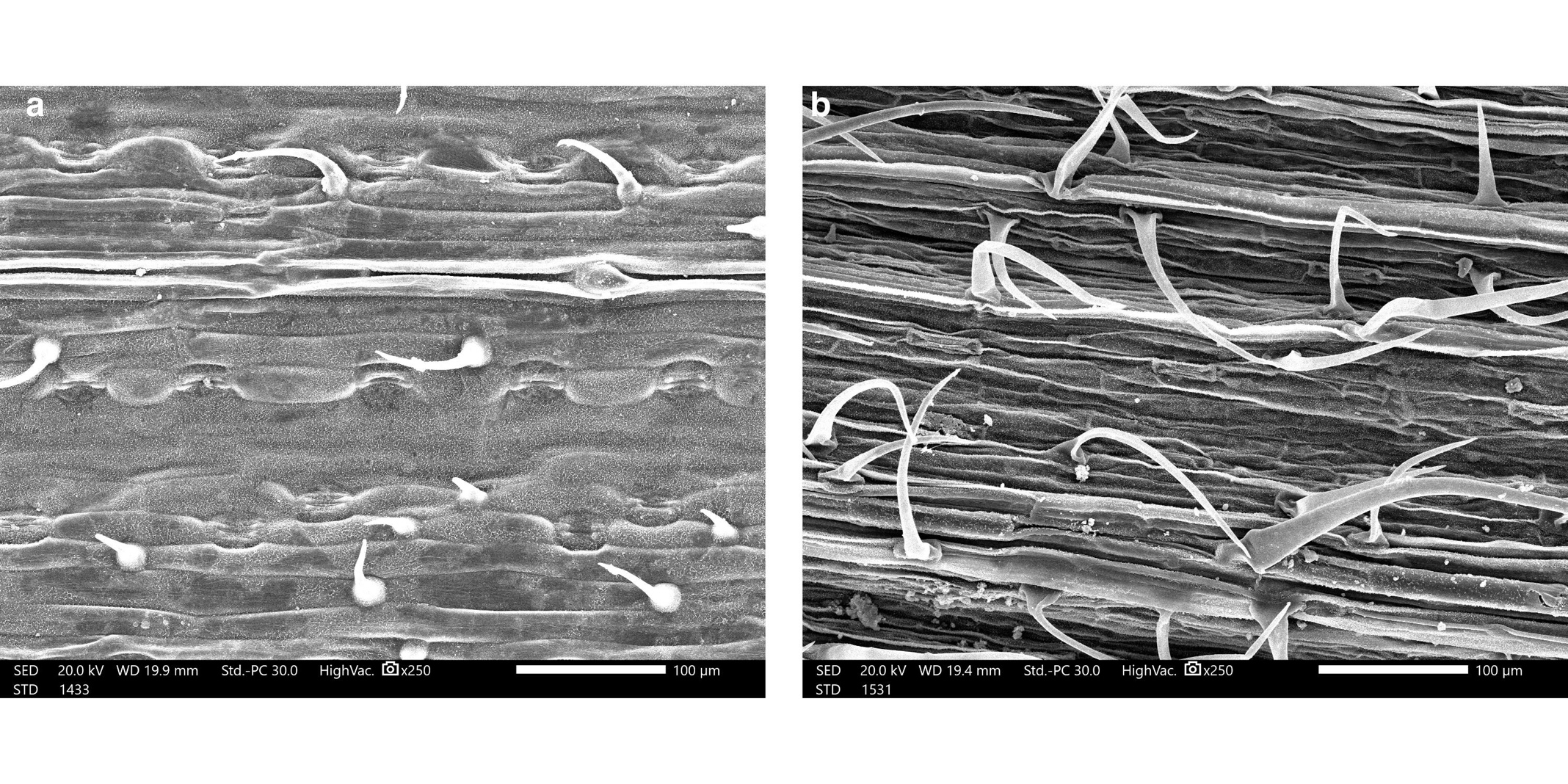


**Figure S1. Scanning electron micrographs of epicuticular wax and trichomes in *Triticum aestivum*.** (a) Control and (b) drought-stressed leaves showing increased wax deposition and prominent trichome development under drought stress. Representative images from SEM analysis.


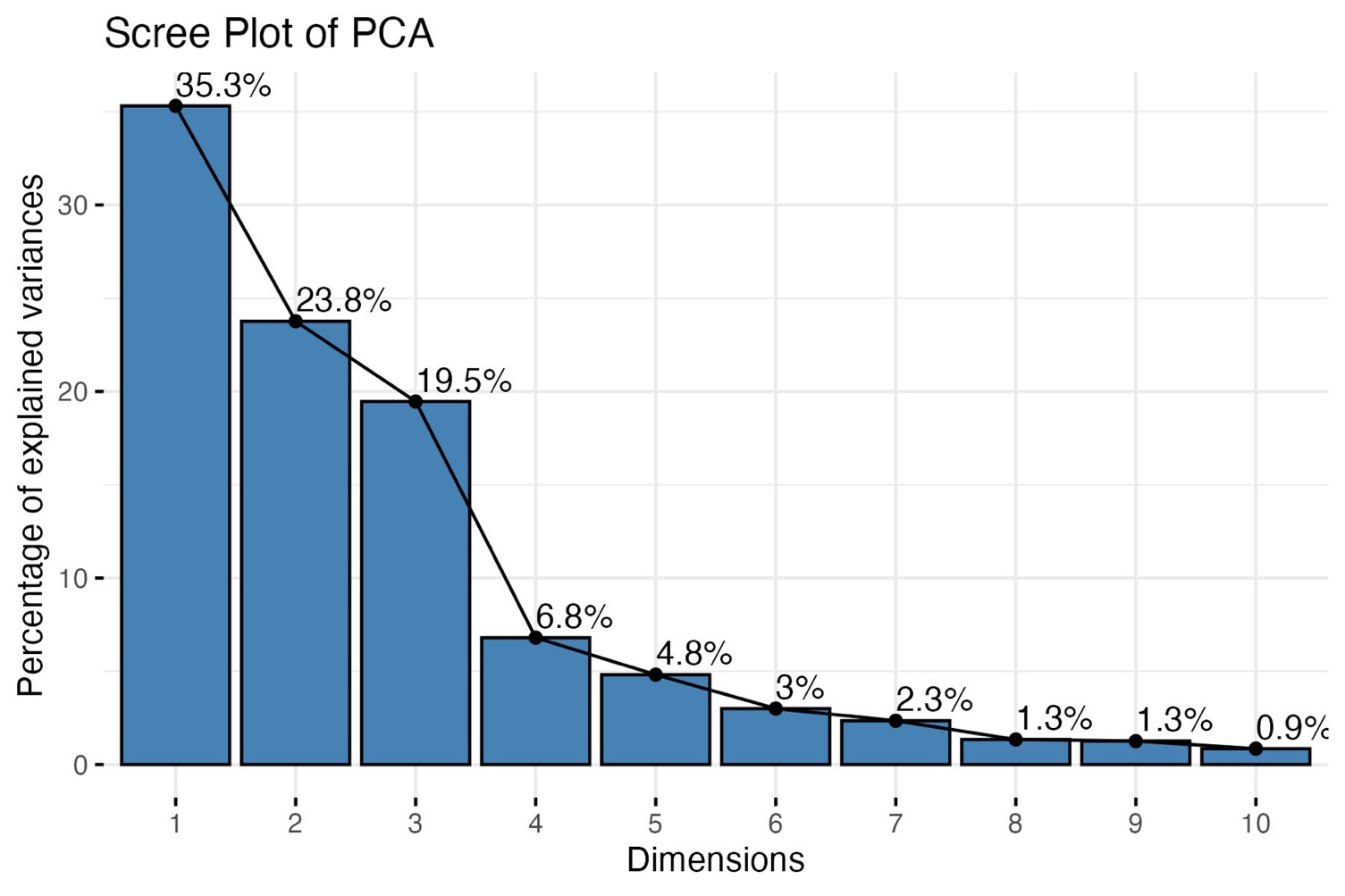


**Figure S2. Scree Plot Showing the Eigenvalues and Percentage of Variance Explained by Each Principal Component.**
